# Fully resorbable poly-4-hydroxybutyrate (P4HB) mesh for soft tissue repair and reconstruction: A scoping review

**DOI:** 10.3389/fsurg.2023.1157661

**Published:** 2023-04-12

**Authors:** Corey R. Deeken, David C. Chen, Manuel Lopez-Cano, David P. Martin, Amit Badhwar

**Affiliations:** ^1^ Covalent Bio, LLC, St. Louis, MO, United States; ^2^ Section of Minimally Invasive Surgery, David Geffen School of Medicine at University of California, Los Angeles, Santa Monica, CA, United States; ^3^ Hospital Universitario Vall d'Hebrón, Universidad Autónoma de Barcelona, Barcelona, Spain; ^4^ Becton, Dickinson and Company, Warwick, RI, United States

**Keywords:** hernia recurrence, P4HB, Phasix Mesh, poly-4-hydroxybutyrate (P4HB), resorbable, surgical site infection (SSI)

## Abstract

**Background:**

Poly-4-hydroxybutyrate (P4HB) is a fully resorbable, biologically-produced polymer with a strength and flexibility comparable to permanent synthetic polymers. The objective was to identify/summarize all peer-reviewed publications involving P4HB mesh.

**Methods:**

A scoping review was conducted within PubMed and included articles published through October 2022.

**Results:**

A total of *n* = 79 studies were identified (*n* = 12 *in vitro*/bench; *n* = 14 preclinical; *n* = 6 commentaries; *n* = 50 clinical). Of the clinical studies, *n* = 40 reported results applicable to hernia and *n* = 10 to plastic/reconstructive surgery and involved patients of all Centers for Disease Control (CDC) wound classes and Ventral Hernia Working Group (VHWG) grades.

**Conclusion:**

P4HB mesh provides long-term hernia repair strength and exhibits promising clinical outcomes beyond its resorption period. Future studies should include randomized controlled trials comparing P4HB to other biomaterials, as well as optimal patient selection, operative technique, long-term outcomes, minimization of potential mesh-related complications, and potential contraindications/complications for P4HB in hernia/abdominal wall reconstruction.

## 1. Introduction

The development of a unique, fully resorbable, biologically-produced, thermoplastic polyester called poly-4-hydroxybutyrate (P4HB) was first reported in 2003 for implantable medical device applications (TephaFLEX®, Tepha Inc., Lexington, Massachusetts) (1). While the chemical synthesis of P4HB is possible (2–4), the molecular weight needed for practical applications as an implantable fiber or mesh can presently only be achieved by fermentation, and there are currently no chemically synthesized P4HB-based products used in commercial medical products or devices. Rather, P4HB is typically produced through a biologic recombinant fermentation process using *Escherichia coli* K12, a microorganism widely utilized in the biopharmaceutical industry to develop other products for human use (1, 5–7). Thus, P4HB is free of the residual metal catalysts that are common in chemically-derived polymers (1, 7). The resulting P4HB is extracted from the fermented cells, purified and processed into fibers using established plastics processing techniques (1, 5, 8).

P4HB is a strong and flexible material, with a tensile strength comparable to permanent synthetic polymers such as polypropylene and ultrahigh molecular weight polyethylene and can be stretched up to 10× its initial length prior to failure (1, 6, 7). P4HB can also be tailored to achieve a wide range of physical and mechanical properties as desired for various applications (1). P4HB has a long-term degradation profile of 12–18 months (5, 9) and degrades into 4HB fragments through bulk hydrolysis, as well as surface erosion (5, 7). With a half-life of approximately 30 min, 4HB degradation products are quickly metabolized through the Krebs cycle, and eliminated as carbon dioxide and water (1, 5, 7). The gradual resorption process results in a steady decline in mechanical strength for a P4HB mesh as the load is progressively transferred back to the repaired tissue (1, 6). This is a major benefit compared to many other resorbable polymers that degrade rapidly through bulk hydrolysis, resulting in a steep decline in mechanical strength before the wound has had time to sufficiently remodel (1, 7).

The United States Food and Drug Administration (FDA) cleared monofilament P4HB suture and monofilament P4HB surgical mesh for human use in 2007, followed by regulatory clearance in Europe in 2009 (5). P4HB has subsequently been utilized to develop a variety of commercially-available devices, including: sutures for tissue approximation (MonoMax® Suture, Aesculap AG, Tuttlingen, Germany), scaffolds to support tendon and ligament repair (BioFiber™ Scaffold, Tornier, Inc., Edina, MN), biomaterials for plastic and reconstructive surgery (GalaFLEX® Scaffold, Galatea Surgical, Inc., Lexington, MA), and meshes for hernia repair (Phasix™ Mesh, Becton, Dickinson, and Company, Franklin Lakes, NJ) (1, 5–7). Currently, P4HB mesh is available in two configurations for hernia repair: as a bare, macroporous mesh (Phasix™ Mesh) or combined with a resorbable hydrogel layer (Phasix™ ST Mesh). The hydrogel layer, comprised of sodium hyaluronate (HA), carboxymethylcellulose (CMC), and polyethylene glycol (PEG) (9), serves as a barrier that minimizes tissue adherence of the bowels to the underlying mesh by separating the abdominal viscera from the P4HB mesh structure (10). The uncoated side of the mesh is porous to allow tissue ingrowth the abdominal wall. The hydrogel is fully resorbed in approximately 30 days (10). The addition of this hydrogel barrier layer to P4HB mesh provides a fully resorbable, barrier mesh option appropriate for intraperitoneal placement (11, 12).

In the last decade, P4HB mesh has been clinically studied in the scientific literature across several surgical specialties (7). In the area of plastic and reconstructive surgery, P4HB mesh has been used primarily in breast surgery (13–21), with some early work in rhytidectomy (16, 22). P4HB mesh has also been evaluated as a potential biomaterial for the repair of pelvic organ prolapse in urogynecological surgery (23–26). The majority of data and clinical research using P4HB mesh has focused on general surgery applications, particularly in the area of hernia repair (i.e., ventral/incisional hernia, incisional hernia prophylaxis, inguinal hernia, and hiatal/paraesophageal hernia) (27–31). The objective of the current study was to review the results of all peer-reviewed studies involving P4HB mesh, guided by the following research questions:

(1) How has P4HB mesh been evaluated to date (i.e., benchtop, preclinical, and clinical peer-reviewed studies)?
(2) What is the clinical role of P4HB mesh in hernia repair, abdominal wall surgery, and plastic/reconstructive surgery?
(3) What are the knowledge gaps and opportunities for future research for P4HB?

## 2. Methods

A scoping review of the published literature was conducted according to the PRISMA-ScR Guidelines (Preferred Reporting Items for Systematic Reviews and Meta-Analyses—extension for Scoping Reviews) (32). A search string with Boolean operators was utilized to retrieve results within the PubMed database, along with a hand search of references from eligible articles, reviews on the topic that were not identified in the literature search, and personal reference collections. Search terms included: “poly-4-hydroxybutyrate” OR “4-hydroxybutyrate mesh” OR “P4HB” OR “Phasix” + “hernia” and included articles published through October 2022. Inclusion criteria included: all peer-reviewed papers involving P4HB mesh, that were published in the English language, and which could be obtained as a full-text. Abstracts, posters, slide presentations, and Letters to the Editor were excluded, as well as articles in which 4HB was combined with other monomers to form a copolymer; P4HB was used as a coating material rather than as a mesh construct; or those articles in which it was determined to be a polymer other than P4HB after review of the full-text.

Outcomes of interest were recorded in a tabular format. Studies were grouped according to category (i.e., bench, preclinical, clinical, commentary). Publication date, mesh type, summary of results, and citation were recorded for all study categories. For bench studies, the type of study and its objective, the technique used, and the results were collected. For preclinical studies in animals, the species used, the surgical technique, the mesh evaluated, and the implantation time were recorded. For clinical studies, the number of patients in the trial, as well as details of the surgical technique and study design were recorded. Additionally, follow-up period and clinical outcomes such as quality of life metrics and rate of hernia recurrence, surgical site infection (SSI), and reoperation were captured and tabulated. Centers for Disease Control (CDC) wound classification (33) and Ventral Hernia Working Group (VHWG) grades (34, 35) were likewise reported. Studies were classified as “restricted” if the study design limited the inclusion of patients with specific CDC wound classification or VHWG grades based on either the original (31) or modified (32) grading systems. Similarly, studies were classified as “unrestricted” if the study design permitted the inclusion of all CDC wound classes and VHWG grades.

## 3. Results

**Figure 1:**
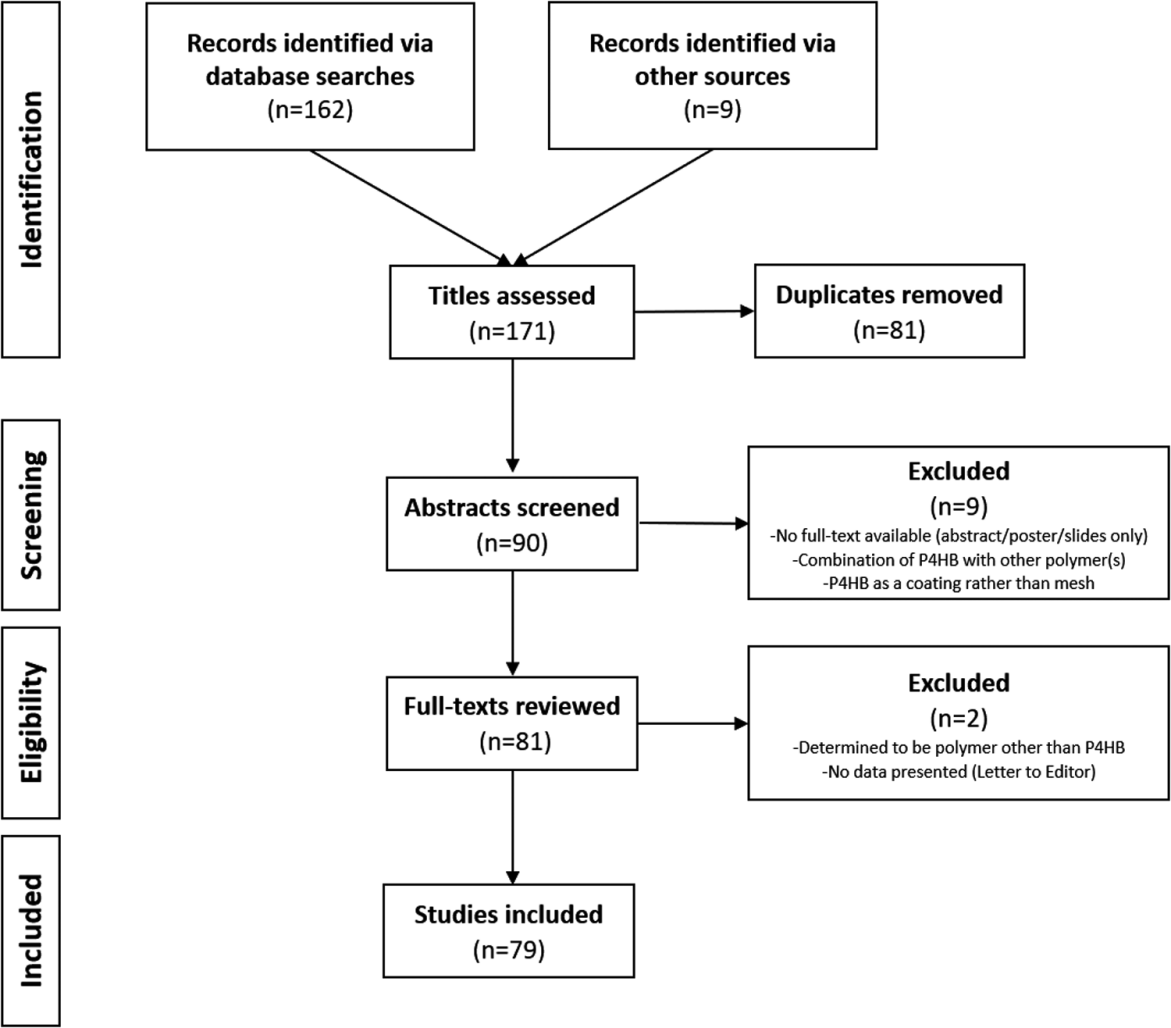
PRISMA-ScR diagram—preferred reporting items for systematic reviews and meta-analyses extension for scoping reviews.

**Figure 2:**
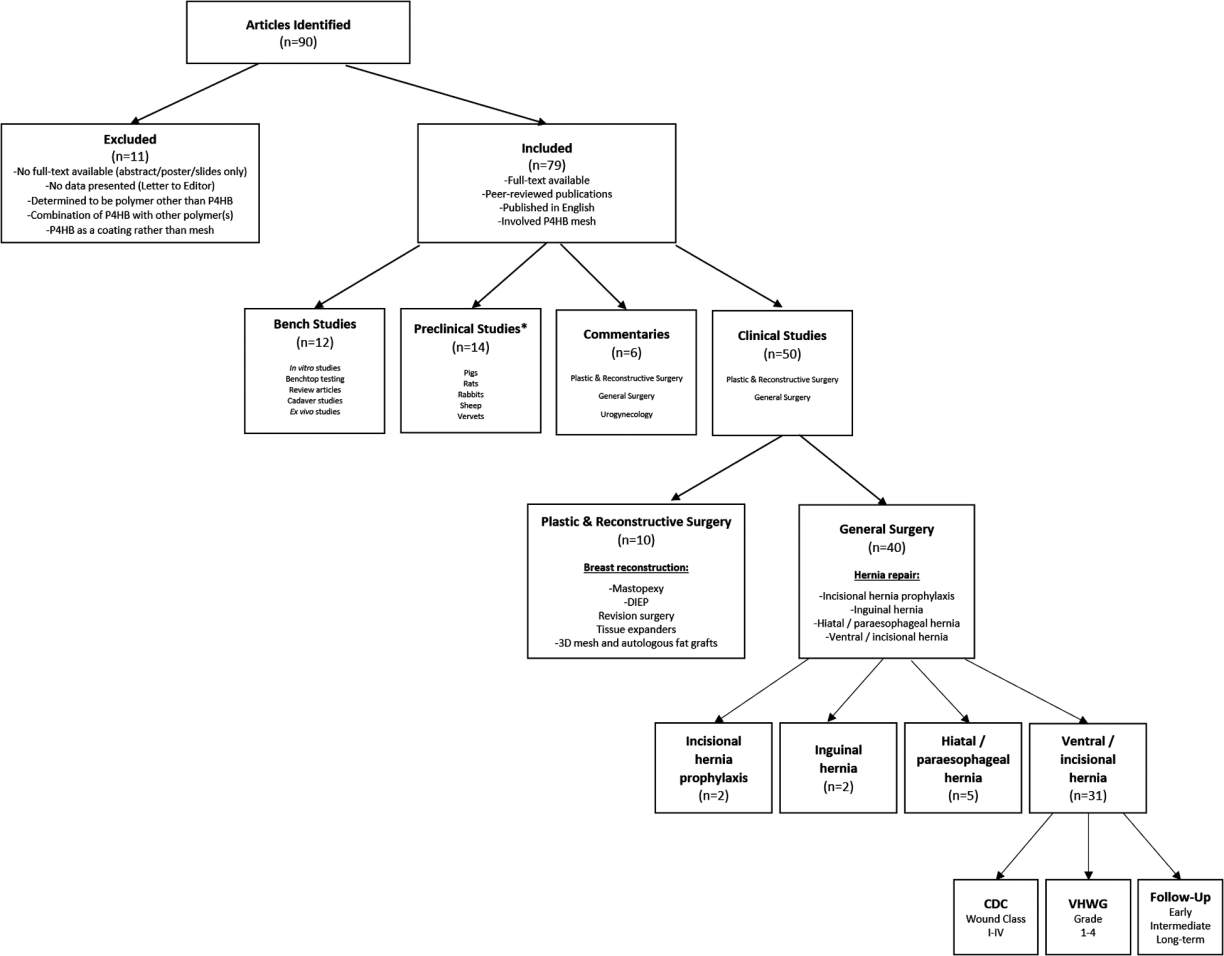
Flowchart depicting depth and breadth of studies that have evaluated P4HB mesh. *n = 3 articles with both Bench and Preclinical components. DIEP, deep inferior epigastric perforator flap.

Following the PRISMA-ScR schematic (Figure 1), a total of *n* = 79 full-text, peer-reviewed publications were identified that involved P4HB mesh and were published in the English language. The articles were subdivided into type of study—bench (*n* = 12), preclinical (*n* = 14), commentaries (*n* = 6), and clinical (*n* = 50) in Figure 2 and Table 1. The clinical studies were further subdivided into plastic and reconstructive surgery (*n* = 10) and general surgery (*n* = 40). The general surgery population was then divided into incisional hernia prophylaxis (*n* = 2) and hernia repair based on type: inguinal hernia (*n* = 2), hiatal/paraesophageal hernia (*n* = 5), and ventral/incisional hernia (*n* = 31). Lastly, all ventral/incisional hernias were stratified, when possible, by CDC wound class, VHWG grade, and/or follow-up period. All published studies are summarized in Table 1, including commentaries comprised of plastic and reconstructive surgery (16, 17, 87), general surgery (86, 88), and urogynecology (25). Table 2 summarizes the clinical outcomes associated with P4HB mesh in incisional hernia prophylaxis, inguinal hernia repair, and hiatal/paraesophageal hernia repair. Table 3 summarizes the clinical outcomes associated with ventral/incisional hernia repair. Studies without specified follow-up time, case reports, and protocols without outcomes data were not included in Tables 3 or 4, but are reported in Table 1 for completeness.

**Table 1:** Study design parameters and outcomes associated with all published studies involving P4HB mesh.

Author (Year)	Type of Study	Technique	Meshes Evaluated	Summary of Results		Ref
**Bench Studies**						
Martin (2013)	Bench	Accelerated degradation	Phasix™ Mesh Bard Mesh	Significant reduction in molecular weight and mechanical strength over time; correlation between strength and fiber integrity	(6)
Williams (2013)	Review	Tissue repair and regeneration	P4HB	Review of FDA cleared devices comprised of P4HB; presentation of mechanical properties and other characteristics of P4HB; discussion of devices currently under development and related patents	(5)
Angelos (2014)	Cadaver	Rhytidectomy (facelift)	P4HB	Superficial musculoaponeurotic system (SMAS) tissue reinforced with P4HB demonstrated significantly improved mechanical properties compared to SMAS without reinforcement.	(22)
Williams (2016)	Review	Plastic & Reconstructive Surgery	GalaFLEX (P4HB)	P4HB fibers are associated with high initial mechanical strength, encourage tissue ingrowth, and offer a long resorption period, leading to a durable repair. Potential applications in rhytidectomy and breast reconstruction are discussed.	(7)
Pineda Molina (2019, AMP)	*In vitro*	Murine bone marrow-derived macrophages	Phasix monomer (4HB)	4HB monomer was not cytotoxic, exhibited increased expression of cathelicidin LL-37 [i.e., an antimicrobial peptide (AMP), *cramp*], and induced transcriptional activation of the AMP *β-defensin-4* in murine bone marrow-derived macrophages; 4HB promoted bacterial resistance through upregulation of antimicrobial peptides (AMPs); 4HB could be used in drug delivery or to functionalize the surface of biomaterials to protect against bacterial contamination.	(36)
Pineda Molina (2019, M1/M2)	*In vitro*	Murine bone marrow-derived macrophages (naïve or pre-activated)	Phasix monomer (4HB)	Phasix™ Mesh degradation products (4HB) significantly decreased cellular expression of the pan macrophage marker F4/80 and significantly increased cellular expression of the M2 macrophage markers Fizzz1 and Arginase1.	(37)
Trapani (2020)	*Ex vivo* model	Mesh embedded in human abdominal fascia tissue	Phasix™ Mesh Polypropylene Control (no mesh)	After 54 days of culture, both meshes displayed disorganized collagen. Phasix™ Mesh exhibited a higher collagen I : III ratio than polypropylene mesh and the control group (no mesh), with favorable mechanical properties.	(38)
Verhorstert (2020)	*In vitro*	Bacterial adherence and biofilm formation	P4HB mesh Polypropylene	Despite greater surface area of P4HB meshes compared to polypropylene meshes, P4HB did not exhibit greater bacterial adhesion or biofilm formation.	(39)
Diedrich (2021)	*In vitro*	Fibroblast attachment and proliferation	P4HB mesh Polypropylee	P4HB exhibited improved cellular attachment and proliferation and significantly greater collagen deposition at 28 days; Knit design parameters could potentially influence/optimize cellular behavior.	(40)
Mora-Navarro (2021)	*In vitro*	Human peripheral blood-derived macrophages	GalaFLEX (P4HB) Prolene Vicryl Mersilene TIGR SERI AlloDerm	Human peripheral blood-derived macrophages exposed to P4HB mesh *in vitro*, co-expressed genes associated with inflammatory, non-inflammatory, and pathogen response phenotypes, suggesting that exposure to P4HB may provoke a unique polarization state.	(41)
Pineda Molina (2021)	*In vitro*	Murine bone marrow-derived macrophages (naïve or pre-activated)	Phasix monomer (4HB)	The results of this study identified the molecular mechanism responsible for transcriptional activation of the antimicrobial peptide cathelicidin LL-37 (*Cramp)*. Upregulation of the *Cramp* gene occurred *via* modulation of GPR109A, a G-protein coupled receptor, and was independent of histone deacetylase (HDAC) activity.	(42)
Singh (2021)	Bench	Low-temperature spinning process	P4HB filaments	Development of wet-spinning process to produce P4HB fibers with a variety of morphologies, mechanical properties, and melting properties; A number of polymer concentrations, bath temperatures, and spin ratios were evaluated.	(8)
Author (Year)	Species	Surgical Technique	Meshes Evaluated	Implantation Time	Summary of Results	Ref.
**Preclinical Studies**						
Martin (2013)	Pig	Repair of a full fascial anterior abdominal wall defect	Phasix™ Mesh	0, 8, 16, 32, 48, 72 weeks	At 72 weeks post-implantation, Phasix™ Mesh-repaired sites demonstrated significantly reduced molecular weight of the P4HB polymer, while maintaining comparable mechanical strength as the native abdominal wall, suggesting that Phasix™ Mesh provides a durable repair.	(6)
Deeken (2013)	Pig	Repair of bilateral abdominal wall defect	Phasix™ Mesh	6, 12, 26, 52 weeks	The burst strength of porcine abdominal wall tissue repaired with Phasix™ Mesh remained stable, with no loss of strength throughout the implantation period. Significant material resorption was documented *via* significant reduction in molecular weight over time. The burst strength of the Phasix™ Mesh-repaired sites remained significantly higher than the native abdominal wall, suggesting successful augmentation of the repair over the 52-week implantation period, despite ongoing resorption. A favorable mild-moderate granulation tissue/neovascularization and mild inflammatory response were also observed.	(43)
Scott (2016)	Pig	Bilateral repair of abdominal wall defects	Retromuscular: Phasix™ Mesh Strattice Intraperitoneal: Phasix™ ST Ventralight ST	12 and 24 weeks	The burst strengths of porcine abdominal wall tissues repaired Phasix™ ST, Ventralight ST, and Phasix™ meshes remained stable, with no loss of strength throughout the 24- week implantation period. All mesh-repaired sites exhibited burst strengths significantly greater than the native abdominal wall, suggesting successful augmentation of the repair sites over the 24-week implantation period. In contrast, the Strattice-repaired sites exhibited a significant decrease in burst strength in the first 12 weeks. Although Strattice-repaired sites exhibited burst strengths significantly higher than the NAW, Phasix™-repaired sites were significantly stronger than Strattice-repaired sites at both 12 and 24 weeks postimplantation.	(44)
Stoikes (2017)	Rat Rabbit	SubQ SubQ w/bacteria	Phasix™ Mesh Bio-A	2, 4, 8, 12, 16, 24 weeks 7 days	Phasix™ Mesh exhibited greater mechanical strength, along with lower abscess scores, bacterial colonization, and inflammation compared to Bio-A.	(45)
Lake (2019)	Rabbit	Bilateral subQ pockets inoculated with MRSA	Phasix™ Mesh Synecor Zenapro Ovitex Permanent Ovitex Resorbable	7 days	Three out of four of the hybrid meshes evaluated exhibited significant bacterial colonization at 7 days postimplantation/inoculation. In contrast, Phasix™ Mesh exhibited a favorable response to bacterial inoculation, with abscess scores of zero, negative pocket swabs, and zero cases of positive bacterial colonization.	(44)
Miserez (2019)	Review of 11 studies: rats, sheep, rabbits, pigs, vervets	Various	Phasix™ Mesh Bio-A TIGR Matrix	Various	Systematic review of the physicochemical characteristics and biomechanical, histological, and clinical outcomes associated with resorbable meshes in experimental preclinical studies.	(46)
Pineda Molina (2019, AMP)	Rat	Bilateral partial thickness abdominal defect AND SubQ dorsal mesh implantation with bacterial contamination	Phasix™ Mesh Polypropylene	3, 7, 14, 21 & 35 days	Both Phasix™ and polypropylene meshes were associated with increased expression of the AMP *cramp* (i.e., cathelicidin LL-37), which was the highest in the Phasix™ mesh group. After deliberate bacterial contamination, polypropylene meshes exhibited significantly greater *S*. *aureus* colonies compared to Phasix™ Mesh, suggesting that the 4HB monomer resists bacterial contamination.	(36)
Pineda Molina (2019, M1/M2)	Rat	Bilateral partial thickness abdominal defect	Bard Mesh TIGR Bio-A Phasix™ Mesh Strattice	3, 7, 14, 21 & 35 days	Phasix™ Mesh activated macrophages from a pro-inflammatory phenotype (M1) to a pro-remodeling phenotype (M2) earlier in the postoperative period than other meshes evaluated and demonstrated favorable tissue remodeling characteristics at 35 days postimplantation. In comparison, the response produced by Bard Mesh, TIGR, Bio-A, and Strattice were all dominated by the M1 pro-inflammatory macrophage phenotype.	(37)
Deeken (2020)	Pig	Ventral hernia repair: 4 and 8 cm unclosed & 8 cm closed defects	Phasix™ ST	48 and 72 weeks	Phasix™ ST Mesh remained intact at 48 weeks, but degraded into fragments by 72 weeks. Mature collagen/fibrovascular tissue was observed around and within the mesh pores. Similar mechanics, degradation, and histological results were observed regardless of defect size or closure technique.	(47)
Pascual (2020)	Rabbit	Partial abdominal wall defects	Phasix™ Mesh Protexa	3, 6, 12, 18 months	Phasix™ Mesh was associated with fewer macrophages than Protexa at the later time points, but many of the other outcomes were similar between meshes (collagen ingrowth, implant area, mechanics).	(48)
Diedrich (2021)	Sheep	Implantation in the posterior vaginal wall	P4HB Polypropylene	60 and 180 days	P4HB mesh was associated with increased M2/M1 ratio, evidence of densely packed collagen fibers, and low myofibroblast differentiation, suggesting favorable tissue response and similar load-bearing compared to polypropylene mesh.	(23)
O-Shaughnessy (2021)	Rabbit	SubQ and peri-vaginal	Phasix™ Mesh Polypropylene	3 and 9 months	Phasix™ Mesh and permanent polypropylene mesh exhibited similar mechanical strength and histological results at both time points, suggesting that Phasix Mesh may be a feasible alternative for pelvic organ prolapse repair.	(24)
Pascual (2021)	Rabbit	Mesh implantation on the intact parietal peritoneum	Phasix™ ST Symbotex Optilene	3, 7, 14 and 90 days	At 90 days postimplantation, Phasix™ Meshes were associated with greater M2 macrophage expression and fewer macrophages overall compared to Symbotex. The neoperitoneum associated with Phasix™ Mesh was significantly thicker than that of the other meshes and was comprised of mature, organized tissue.	(49)
Verhorstert (2022)	Mouse	Subcutaneous implantation of mesh in infection model with intentional bacterial inoculation	P4HB Polypropylene	4, 9, 60 days	Host tissue response, clearance of bacteria, and adverse events were similar for P4HB and polypropylene meshes despite the larger surface area associated with P4HB mesh.	(26)
Author (Year)	Type of Study	Surgical Technique	Meshes Evaluated	Number of Patients	Summary of Results	Ref
**Clinical Studies**						
Adams (2017)	Prospective, Single center	Primary mastopexy with soft tissue reinforcement	GalaFLEX (P4HB)	11	This study showed promising results when GalaFlex (P4HB) was used to reinforce the lower pole of the breast in *n* = 11 consecutive patients. The lower pole stretched 5% over 12 months.	(13)
Buell (2017)	Retrospective review	Complex abdominal wall reconstruction with primary fascial closure & mesh overlay	Strattice: *n* = 42 Phasix™: *n* = 31	73	Phasix™ Mesh exhibited significantly fewer complications and recurrences, with a significant cost savings relative to Strattice.	(50)
Wormer (2017)	Retrospective review	Deep inferior epigastric perforator (DIEP) flap breast reconstruction with onlay mesh	Phasix™ *n* = 160 No mesh *n* = 159	319	Phasix™ Mesh may improve cosmesis (reduce postoperative bulge) following DIEP reconstruction, with similar donor site complications as repairs completed without mesh reinforcement.	(14)
Adams (2018)	Prospective, multicenter	Elective mastopexy and reduction mammaplasty	GalaFLEX (P4HB)	62	GalaFLEX successfully corrected ptosis and maintained that correction at 1 year after implantation in 89.7% of cases.	(18)
Plymale (2018)	Prospective pilot study	Ventral and incisional hernia repair; Rives–Stoppa approach with retrorectus mesh placement	Phasix™ Mesh	31	At 24-months follow-up, there were no hernia recurrences or infections, and quality of life was significantly improved over baseline; 19.4% of patients experienced an adverse event (e.g., seroma, wound necrosis, and wound dehiscence).	(51)
Roth (2018)	Prospective, multicenter	Retrorectus or onlay VIHR	Phasix™ Mesh	121	At 18-months follow-up, patients at high risk for postoperative complications experienced low rates of hernia recurrence (9%), SSI (9%), seroma (6%), reoperation (8%), and adverse events (9%) when repaired with Phasix™ Mesh.	(52)
van Rooijen (2018)	Prospective, multicenter	VHWG Grade 3 midline incisional hernia repair using (retrorectus or onlay)	Phasix™ Mesh	85	Protocol for upcoming clinical trial; no results presented; Primary outcome = surgical site occurrence (SSO) through 3 months postimplantation Secondary outcomes = recurrence, infection, and quality of life through 24 months postimplantation	(53)
Kniepeiss (2019)	Randomized control trial	Prophylactic onlay mesh placement to prevent incisional hernia repair after liver transplantation	Phasix™ Mesh No mesh	194	Protocol for upcoming clinical trial; no results presented; Primary outcome = incisional hernia at 12 months post-implantation Secondary outcome = incisional hernia at 24 months post-implantation and rate of mesh-related complications (i.e., hematoma, seroma, pain, wound dehiscence, infection, mesh removal)	(54)
Messa (2019)	Retrospective review	Complex VHR	Phasix™ Mesh	70	24-months after complex VHR, Phasix™ Mesh demonstrated significant improvement in quality-of-life metrics, low hernia recurrence rates (5.7%), and favorable clinical outcomes (low rates of seroma and wound dehiscence and no cases of mesh infection or excision).	(55)
Nair (2019)	Retrospective review	Complex breast revision	GalaFLEX (P4HB)	5	GalaFLEX demonstrated favorable aesthetic outcomes when used to prevent sagging of the lower pole in breast reconstruction procedures, with improvement in Baker Grade Contraction scores.	(56)
Abdelmoaty (2020)	Retrospective review of prospective database	Laparoscopic repair of paraesophageal hernia	Phasix™ ST	50	This study demonstrates the safety and efficacy of Phasix™ ST Mesh for crural reinforcement at the hiatus over the short-term (median: 12 months follow-up); No major complications were reported (mesh infection or erosion); Recurrence rate (8%) and reoperation rate (0%) were low.	(57)
Aiolfi (2020)	Retrospective single-center review	Laparoscopic posterior cruroplasty for hiatal hernia repair	No mesh (*n* = 102) Phasix™ ST (*n* = 39)	141	Low rates of complications (4.2%) and recurrences (2.1%) were observed, along with improved quality of life metrics, but the results were not subdivided into mesh-based repairs versus suture repair without mesh reinforcement.	(58)
Aldohayan (2020)	Prospective pilot study	Laparoscopic TAPP inguinal hernia repair	Phasix™ Mesh	15	Phasix™ Mesh performed well in this pilot study, with no hernia recurrences (0%) and very limited chronic pain in inguinal hernias repaired *via* laparoscopic TAPP technique.	(28)
Calobrace (2020)	Retrospective review	Popcorn capsulorrhaphy in revision aesthetic breast surgery	GalaFLEX (P4HB)	149	The technique of popcorn capsulorrhaphy can improve control and stability of the pocket in aesthetic breast surgery. The use of a mesh such as GalaFLEX may reduce complications and revisions.	(19)
Levy (2020)	Retrospective review	Tissue expander-based breast reconstruction after mastectomy	Phasix™ (*n* = 62) AlloMax (107)	169	Phasix™ Mesh was associated with significantly shorter drain duration compared to AlloMax, with comparable incidence of infection, necrosis, seroma, and reoperation.	(15)
Mellia (2020)	Review of previous literature	Ventral hernia repair	Phasix™ Mesh	453	Systematic review of the literature showed low rates of SSI (6.8%), reoperation (10.7%) and hernia recurrence (9.1%); Onlay repairs were associated with significantly higher rate of recurrences compared to sublay.	(59)
Pakula (2020)	Retrospective review	Complex ventral hernia repair with retromuscular mesh	Phasix™ Mesh	20	At a mean follow-up of 21.1 months following implantation of Phasix™ Mesh, low rate of recurrence (0%), SSI (10%), and seroma (10%) were observed in this cohort of high-risk patients.	(60)
Panici Tonucci (2020)	Single-center observational cohort study	Laparoscopic repair of hiatal hernia reinforced with mesh	Phasix™ ST	73	At a median follow-up of 17 months, Phasix™ ST Mesh was shown to be safe and effective for reinforcing crural repairs at the hiatus (3.2% recurrence and 0% mesh-related complications, with significantly improved quality of life metrics and no reoperations required).	(29)
Rehnke (2020)	Retrospective review	Breast reconstruction with 3-D mesh and autologous fat grafting	Lotus scaffold: comprised of Phasix™ Mesh, TIGR Matrix, or SERI	22	At a mean follow-up of 19 months, adipose tissue was observed around the Lotus scaffold. No capsule formation, oil cysts, or calcification was observed, and all patients were satisfied with the cosmetic outcome (i.e., size and shape of reconstructed breast).	(61)
Rognoni (2020)	Observational, prospective, multi-center study	Primary ventral or incisional hernia repair	Phasix™ Mesh Phasix™ ST	75	Phasix™ and Phasix™ ST Mesh demonstrated promising outcomes with low rates of recurrences (8.0%) and complications (1.3% infected mesh removal; 4.0% SSI with intervention, 6.7% seroma with intervention, and 5.3% reoperation) and significantly improved quality of life metrics.	(62)
van Rooijen (2020)	Prospective, multi-center single-arm study	VHWG Grade 3 midline incisional hernia repair (potentially contaminated)	Phasix™ Mesh	84	At short-term follow-up of 3 months, encouraging results were reported when Phasix™ Mesh was used to repair potentially contaminated incisional hernias (0% hernia recurrence, 13% SSI).	(63)
Yu (2020)	Retrospective review	Bilateral free flap breast reconstruction	Phasix™ *n* = 40 Polypropylene *n* = 20 Primary closure *n* = 6	66	Though more expensive than polypropylene or primary repair, Phasix™ Mesh successfully reinforced the abdominal wall, leading to significantly fewer complications compared to polypropylene mesh (Phasix™ Mesh: 0% seroma, 0% wound dehiscence, 0% reoperation, and 0% hernia) (Polypropylene mesh: 10% seroma, 20% wound dehiscence, 10% reoperation, and 10% hernia)	(64)
Aldohayan (2021)	Retrospective review	Laparoscopic ventral/incisional hernia repair	Phasix™ ST	26	At a mean follow-up of 28 months following implantation of Phasix™ ST Mesh, 0% hernia recurrence and 0% wound infection were reported, with pain levels consistently decreasing over time.	(65)
Buell (2021)	Retrospective cohort study	Complex abdominal wall reconstruction	Phasix™ *n* = 31 Strattice *n* = 42	73	At 5 year follow-up, Phasix™ Mesh exhibited significantly improved clinical outcomes (complications, recurrences, infections) and significant cost-savings compared to porcine dermal matrices (Strattice).	(30)
Bueno-Lledo (2021, Abd)	Retrospective review	Replacement of infected mesh with Phasix™ using a one-stage (*n* = 30) or two-stage (*n* = 41) surgical approach	Phasix™ Mesh	71	Phasix™ Mesh was associated with 7% recurrence and 1.4% infection when utilized in single-stage management of chronic mesh infection and may represent a better option than two-stage approach.	(66)
Bueno-Lledo (2021, Bio)	Retrospective review	Single-stage treatment of chronic mesh infection with mesh removal and Phasix™ mesh implantation	Phasix™ Mesh	30	Clinical outcomes were promising nearly three years after implantation of Phasix™ Mesh to replace infected mesh (3.3% recurrence, 3.3% infection).	(67)
Charleux-Muller (2021, Slowly)	Retrospective cohort, multicenter	Incisional hernia repair in contaminated surgical field	Phasix™ *n* = 90 Phasix™ ST *n* = 103	215	At an average follow-up of 11.7 months, Phasix™ and Phasix™ ST repairs in contaminated fields exhibited an overall recurrence rate of 12.4% and SSI of 22.3%	(68)
Charleux-Muller (2021, Cost)	Retrospective analysis	Potentially contaminated hernia repair	Biologic mesh *n* = 52 Phasix™ *n* = 42	94	In a cost-effectiveness model, Phasix™ Mesh was shown to be more clinically effective (fewer serious complications) and less costly than biologic mesh in VHWG Grade 3 hernia repairs.	(69)
Christopher (2021, An Eval)	Retrospective analysis	Ventral hernia repair	Phasix™ Mesh	71	Despite nearly one-third of cases in contaminated settings, Phasix™ Mesh exhibited a reasonable recurrence rate (12.7%) with significant improvement in quality-of-life metrics over time.	(70)
Christopher (2021, Onlay)	Retrospective review	Complex ventral hernia repair	Phasix™ Mesh	51	At a median follow-up of 20.8 months after Phasix™ Mesh implantation during complex VHR, 5.9% patients experienced hernia recurrence and 15.7% experienced an SSI.	(71)
Christopher (2021, Resorb)	Retrospective cohort study	Ventral hernia repair in CDC wound class II–IV (VHWG Grade 3–4)	Phasix™ Mesh	60	At a median follow-up of 24.2 months postimplantation in contaminated settings, Phasix™ Mesh was associated with 8.3% recurrence, 16.7% SSI, and significantly improved quality of life, suggesting an acceptable safety profile in a complex and high-risk population.	(72)
Claessen (2021)	Retrospective study	Open single-stage complex abdominal wall reconstruction	Phasix™ *n* = 40 Bio-A *n* = 30	70	In this complex, comorbid population, patients were at high risk of complications regardless of mesh type. At a median follow-up of 35 months, Phasix™ Mesh was associated with 10% hernia recurrence rate, similar to that of Bio-A (10%, median follow-up: 11 months). Bio-A meshes were salvaged more frequently (100%) than Phasix™ Meshes (58.8%) after direct contact with SSI. The long-term durability of Bio-A remains unknown, and further studies are warranted.	(73)
Faulkner (2021)	Retrospective review	Prophylactic mesh augmentation	Phasix™ Mesh	50	At a median follow-up of 2.2 years, Phasix™ Mesh exhibited promising results when used to prevent incisional hernia formation (4% seroma, 4% SSI, 20% hernia development); no mesh infections observed; longer-term studies are justified	(27)
Lambrecht (2021)	Case report	Conservative treatment of a patient with a heavily infected Phasix mesh	Phasix™ Mesh	1	In this case report, an infected Phasix™ Mesh was successfully treated in a conservative manner without mesh excision, demonstrating some of the potential benefits associated with this material.	(74)
Levy (2021)	Prospective evaluation	Complex abdominal wall reconstruction	Phasix™ Mesh	105	At 36 months follow-up (mean) after complex abdominal wall reconstruction with Phasix™ Mesh, favorable recurrence (17%) infection (5%), and seroma (6%) rates were observed.	(75)
Roth (2021)	Prospective, multicenter study	Retrorectus or onlay VIHR	Phasix™ Mesh	121	At 36-months follow-up, 0% of patients developed late mesh-related complications requiring removal, while 9.3% reported SSI and 17.9% exhibited hernia recurrence.	(76)
van Driel (2021)	Retrospective, single-arm multicenter	Inguinal or ventral hernia or to replace infected synthetic mesh	Phasix™ Mesh Phasix™ ST	47	At short-term follow-up (mean 140 days; median 48 days), mesh excision was required in only *n* = 1 case despite contaminated or dirty conditions at the time of mesh implantation. Conservative management and minor reoperations were required, and further research is warranted.	(11)
van Rooijen (2021)	Prospective multicenter clinical trial	Ventral hernia repair	Phasix™ Mesh	84	Reasonable recurrence (8%) and reoperation (15.5%) rates were observed 24 months after implantation of Phasix™ Mesh in VHWG Grade 3 patients. No significant improvement in pain or quality of life metrics were observed.	(77)
Vauclair (2021)	Prospective evaluation	Incisional hernia repair with sublay or intraperitoneal mesh placement	Phasix™ Mesh	29	At 1 year following implantation of Phasix™ Mesh in potentially contaminated incisional hernias, there were no instances of mesh infection (0%) and reasonable rates of hernia recurrence (10.3%).	(78)
Aiolfi (2022)	Retrospective, single center study	Laparoscopic paraesophageal hernia repair with Toupet fundoplication	Phasix™ ST	68	At a median follow-up of 27 months, Phasix™ ST was associated with 8.8% hernia recurrence, 0% mesh-related complications, 0% reoperation, and significant improvement in quality-of-life metrics over baseline.	(79)
Hope (2022)	Prospective, multicenter	Laparoscopic or robotic VIHR	Phasix™ ST	120	At 24 months postimplantation, Phasix™ ST Mesh was associated with low rates of SSO and mesh-related complications and improved quality-of-life metrics, but recurrence rates were high at 31.7%. Further analysis indicated that recurrence rates were higher in large defects (≥7.1 cm^2^), indicating that Phasix™ ST Mesh may be better suited for small defects until additional studies can further investigate outcomes in laparoscopic IPOM of large defects.	(12)
Konstantinidis (2022)	Retrospective cohort study	Laparoscopic hiatal hernia repair with 360° Nissen fundoplication	Phasix™ ST	30	At a median follow-up of 14 months following repair of large, complicated hiatal hernias, Phasix™ ST Mesh was associated with 0% hernia recurrence and 0% mesh-related complications.	(80)
Layer (2022)	Retrospective, multicenter	Abdominal wall repair with onlay, retromuscular, or intraperitoneal mesh	Phasix™ Mesh Phasix™ ST	108	At a mean follow-up of 41 months following abdominal wall repair with Phasix™ Mesh or Phasix™ ST Mesh in high-risk, contaminated wounds, recurrence rates (22.2%) were lower than biologic mesh in similar populations and comparable to synthetic mesh in the general population.	(81)
Lima (2022)	Retrospective, single center study	Abdominal wall repair with mesh	Phasix™ Mesh Phasix™ ST	51	At a median follow up of 3.5 months (105 days) following abdominal wall repair with Phasix™ Mesh, hernia recurrence was low at 4.1%, and SSI at 30 days was 13.7%	(82)
Mallucci (2022)	Observational study	Aesthetic breast surgery	GalaFLEX (P4HB)	100	GalaFLEX was utilized in *n* = 100 aesthetic breast surgeries in an observational case series. Photographic analysis showed that the lower pole position was stable over a median follow-up period of 14 months, Many secondary defects such as inferior malposition and symmastia were corrected through the use of this biomaterial.	(20)
Othman (2022)	Retrospective, single center study	Abdominal wall reconstruction with onlay resorbable mesh or intraperitoneal biologic mesh	Phasix™ Mesh Biologic Mesh (XenMatrix or Permacol)	88 (*n* = 44/mesh type)	Higher rates of recurrence, complications, and cost were associated with intraperitoneal biologic mesh implantation compared to onlay resorbable synthetic mesh (Phasix™ Mesh).	(83)
Roth (2022)	Prospective, multicenter	Retrorectus or onlay VIHR	Phasix™	121	At 60-months follow-up, comorbid patients undergoing VIHR with Phasix™ Mesh experienced 10.1% SSI, 14.9% reoperation, 22.0% recurrence, and improved quality of life compared to baseline.	(31)
Schecter (2022)	Retrospective, single center study	Single-stage abdominal wall reconstruction in contaminated or dirty/infected wounds	P4HB	34	At a mean follow-up of 37 months following single-stage abdominal wall reconstruction in contaminated (CDC Class III) or dirty/infected (CDC Class IV) wounds, P4HB mesh was associated with low incidence of hernia recurrence (6%), seroma with intervention (12%), and surgical site infection (9%), with no instances of mesh-related adverse events, mesh infection, or mesh explantation.	(84)
Sigalove (2022)	Retrospective, study	Two-stage, expander-implant, prepectoral breast reconstruction	GalaFLEX-AlloDerm AlloDerm	*n* = 135 patients (*n* = 250 breasts) *n* = 128 patients (*n* = 249 breasts)	The addition of a P4HB component (GalaFLEX) to the AlloDerm repair resulted in comparable postoperative complications as repairs completed with AlloDerm alone. Future studies are warranted to evaluate this biomaterial over a long-term period.	(21)
Talwar (2022)	Retrospective, single center study	Retrorectus or onlay VIHR	Phasix™	51	At a median follow-up of 62.3 months, patients undergoing VIHR with Phasix™ Mesh experienced 20% recurrence, 3.9% SSI, 11.8% reoperation, and significantly improved quality of life at all time periods evaluated	(85)

Author (Year)	Summary of Results					Ref
**Commentaries**						
Adams (2016)	A review of the clinical use of P4HB mesh in both facial and breast surgery applications					(16)
Baxter (2016)	A review of the concept of an “internal bra” for reconstructive and aesthetic breast surgery					(17)
Berrevoet (2020)	Commentary on Deeken *et al*. (47)—recommends more clinical trials, including RCT and registries					(86)
Haddock (2020)	Commentary on Rehnke et al. (61)—discussion of the difficulties in ensuring adequate oxygen delivery to support fat graft survival and prevent calcification or formation of cysts					(87)
Rostaminia (2021)	Commentary on O-Shaughnessy *et al*. (24)—recommends P4HB mesh as an alternative to permanent mesh for pelvic organ prolapse repair					(25)
Morales-Conde (2022)	Consensus statements regarding the use of biosynthetic absorbable mesh in VHWG Grades 2–3 hernias					(88)

**Table 2:** Clinical outcomes associated with P4HB mesh in incisional hernia prophylaxis, inguinal hernia repair, and hiatal/paraesophageal hernia repair.

Author (Year)	Type of Study	Surgical Technique	Meshes Evaluated	Number of Patients	Follow-Up (time)	Summary of Results	Ref
**Incisional Hernia Prophylaxis**							
Kniepeiss (2019)	Randomized controlled trial	Prophylactic onlay mesh placement to prevent incisional hernia repair after liver transplant	Phasix™ Mesh No mesh	194	1, 3, 6, 12, 18, 24 months	Protocol for planned clinical trial; No results presented	(54)
Faulkner (2021)	Retrospective review	Prophylactic mesh augmentation	Phasix™ Mesh	50	2.2 years	Seroma = 4% SSI = 4% Mesh infection = 0% Hernia development = 20%	(27)
**Inguinal Hernia Repair**							
Aldohayan (2020)	Prospective pilot study	Laparoscopic TAPP inguinal hernia repair	Phasix™ Mesh	15	1 week 1, 3, 6, 12, 24 months	Hernia recurrence = 0% Pain = 8.3% at 12 months (0% at 24 months)	(28)
van Driel (2021)	Retrospective, Single-arm Multicenter	Inguinal/ventral hernia or to replace infected synthetic mesh	Phasix™ Mesh Phasix™ ST	47	48 days	Hernia recurrence (overall): 12.8% SSI (overall): 23.4%	(11)
**Hiatal**/**Paraesophageal Hernia Repair**							
Abdelmoaty (2020)	Retrospective review of prospective database	Laparoscopic repair of paraesophageal hernia	Phasix™ ST	50	12 months	Hernia recurrence = 8% SSI = 0%	(57)
Aiolfi (2020)	Retrospective single-center review	Laparoscopic posterior cruroplasty for hiatal hernia repair	No mesh *n* = 102 Phasix™ ST *n* = 39	141	21 months	Hernia recurrence = 2.1% Pain = decreased significantly over time	(58)
Panici Tonucci (2020)	Single-center observational cohort study	Laparoscopic repair of hiatal hernia with mesh	Phasix™ ST	73	17 months	Hernia recurrence = 3.2%	(29)
Aiolfi (2022)	Retrospective, single center study	Laparoscopic paraesophageal hernia repair with Toupet fundoplication	Phasix™ ST	68	27 months	Hernia recurrence = 8.8% Mesh-related complications = 0% Reoperation = 0% QoL metrics = improved over baseline	(79)
Konstantinidis (2022)	Retrospective cohort study	Laparoscopic hiatal hernia repair with 360° Nissen fundoplication	Phasix™ ST	30	14 months	Hernia recurrence = 0% Mesh-related complications = 0%	(80)

**Table 3:** Clinical outcomes associated with P4HB mesh in ventral/incisional hernia repair; categorized by follow-up time.

Author (Year)	Type of Study	Patients	Meshes Evaluated	Surgical Technique	Follow-Up (months)	Recurrence (%)	SSI (%)	Re-operation (%)	Quality of Life	Ref
**Short-Term Studies (<2 years follow-up)**										
Roth (2018)	Prospective, multicenter	121	Phasix™	Retrorectus or onlay VIHR	18	9%	9%	8%	Improved over baseline	(52)
Pakula (2020)	Retrospective	20	Phasix™	Complex VIHR with retromuscular mesh placement	21.1	0%	10%	NR	Improved over baseline	(60)
van Rooijen (2020)	Prospective, multicenter	84	Phasix™	Retrorectus or onlay VHR	3	0%	13.1%	NR	NR	(63)
Rognoni (2020)	Prospective, multicenter	75	Phasix™ Phasix™ ST	Primary VIHR	≥18	8%	4%	5.3%	Improved over baseline	(62)
Charleux-Muller (2021, Slowly)	Retrospective cohort, multicenter	215	Phasix™ *n* = 90 Phasix™ ST *n* = 103	Incisional hernia repair in contaminated surgical field	11.7	14.1%	22.3%	NR	NR	(68)
Christopher (2021, Onlay)	Retrospective	51	Phasix™	Complex VIHR	20 (median)	5.9%	15.7%	7.8%	Improved over baseline	(71)
Claessen (2021)	Retrospective	70	Phasix™: *n* = 40 Bio-A: *n* = 30	Open, single-stage complex abdominal wall reconstruction	20 (median)	Phasix™: 10% Bio-A: 10%	Phasix™: 25% Bio-A: 23.3%	Phasix™: 25% Bio-A: 13.3%	NR	(73)
Lima (2022)	Retrospective	51	Phasix™	Abdominal wall repair	3.5 (median)	4.1%	13.7% (at 30 days)	NR	NR	(82)
Vauclair (2021)	Prospective	29	Phasix™	Incisional hernia repair with sublay mesh	12	10.3%	0%	NR	NR	(78)
**Intermediate-Term Studies (2–3 years follow-up)**										
Plymale (2018)	Pilot study	31	Phasix™	Retrorectus VIHR	24	0%	NR	NR	Improved over baseline	(51)
Messa (2019)	Retrospective	70	Phasix™	Retrorectus or onlay VIHR	24	5.7%	8%	11%	Improved over baseline	(55)
Aldohayan (2021)	Retrospective, single center	26	Phasix™ ST	Laparoscopic VIHR	28	0%	0%	NR	NR	(65)
Bueno-Lledo (2021)	Retrospective	30	Phasix™	One-stage treatment of chronic mesh infection with mesh removal and implantation of new mesh	34.5	3.3%	3.3%	NR	NR	(67)
Bueno-Lledo (2021)	Retrospective review	71	Phasix™: *n* = 30 Synthetic: *n* = 41	Replacement of infected mesh with Phasix™ using a one-stage approach or synthetic mesh in a two-stage approach	36	Phasix™: 6.6% Synthetic: 7.2%	Phasix™: 3.3% Synthetic: 9.8%	Phasix™: 0% Synthetic: 7.3%	NR	(66)
Christopher (2021, Resorb)	Retrospective	60	Phasix™	VIHR in CDC wound class II–IV (VHWG grade 3 & 4)	24.2 (median)	8.3%	16.7%	15%	Improved over baseline	(72)
Levy (2021)	Prospective	105	Phasix™	Complex abdominal wall reconstruction with onlay mesh	36	17%	5%	15%	NR	(75)
Roth (2021)	Prospective, multicenter	121	Phasix™	Retrorectus or onlay VIHR	36	17.9%	9.3%	11.6%	Improved over baseline	(76)
van Rooijen (2021)	Prospective, multicenter	84	Phasix™	Retrorectus or onlay VHR	24	11%	13.1%	15.5%	Improved over baseline	(77)
Hope (2022)	Prospective, multicenter	120	Phasix™ ST	Laparoscopic or Robotic VIHR	24	31.7%	0%	18.3%	Improved over baseline	(12)
Othman (2022)	Retrospective, single center	88	Phasix™ (*n* = 44) Biologics (*n* = 44)	Complex abdominal wall reconstruction	24.5 (median)	Phasix™: 4.5% Biologics: 22.7% (*p* < 0.026)	Phasix™: 18.2% Biologics: 25.6% (*p* = 0.45)	NR	NR	(83)
**Long-Term Studies (>3 years follow-up)**										
Buell (2021)	Retrospective	73	Phasix™ (*n* = 31) Strattice™ (*n* = 42)	Complex abdominal wall reconstruction	60	Phasix™: 12.9% Strattice™: 38.1%	Phasix™: 12.9% Strattice™: 31.0%	Phasix™: 10.0% Strattice™: 14.0%	NR	(30)
Christopher (2021)	Retrospective	71	Phasix™	VIHR	43.1 (median)	12.7%	7%	8.5%	Improved over baseline	(70)
Layer (2022)	Retrospective, multicenter	108	Phasix™ Mesh Phasix™ ST	Abdominal wall repair with onlay, retromuscular, or intraperitoneal mesh	41	22.2%	24.1%	NR	NR	(81)
Roth (2022)	Prospective, multicenter	121	Phasix™	Retrorectus or onlay VIHR	60	22.0%	10.1%	14.9%	Improved over baseline	(31)
Schecter (2022)	Retrospective, single center	34	P4HB	Single-stage abdominal wall reconstruction in contaminated or dirty/infected wounds	37	6%	9%	NR	NR	(84)
Talwar (2022)	Retrospective, single center	51	Phasix™	Retrorectus or onlay VIHR	62.3 (median)	20%	3.9%	11.8%	Improved over baseline	(85)

**Table 4:** CDC wound class and ventral hernia working group (VHWG) grade associated with clinical studies involving P4HB mesh in ventral/incisional hernia repair.

Author (Year)	Number of Patients	CDC Wound Class		VHWG Grade^†^^,^^‡^		Ref
		Unrestricted	Restricted	Unrestricted	Restricted	
Buell (2017)	73	X		X		(50)
Plymale (2018)	31		X (Class I & II)	X		(51)
Roth (2018)	121		X (Class I)		X (Grade ≥2)^†^	(52)
Messa (2019)	70	X		X		(55)
Pakula (2020)	20	X		X		(60)
Rognoni (2020)	75	X			X (Grade 2 & 3)^†^	(62)
van Rooijen (2020)	84	X			X (Grade 3)^†^	(63)
Aldohayan (2021)	26	X		X		(65)
Buell (2021)	73	X		X		(30)
Bueno-Lledo (2021, Abd)	71		X (Class IV)	X		(66)
Bueno-Lledo (2021, Bio)	30		X (Class IV)	X		(67)
Charleux-Muller (2021, Slowly)	215		X (Class ≥2)		X (Grade ≥2)^‡^	(68)
Charleux-Muller (2021, Cost)	94	X			X (Grade 3)^‡^	(69)
Christopher (2021, An Eval)	71	X		X		(70)
Christopher (2021, Onlay)	51	X		X		(71)
Christopher (2021, Resorb)	60		X (Class ≥2)	X		(72)
Claessen (2021)	70	X		X		(73)
Levy (2021)	105	X		X		(75)
Roth (2021)	121		X (Class I)		X (Grade ≥2)^†^	(76)
van Rooijen (2021)	84	X			X (Grade 3)^†^	(77)
Vauclair (2021)	29	X		X		(78)
Hope (2022)	120		X (Class I)		X (Grade ≥2)^†^	(12)
Layer (2022)	108		X (Class ≥2)		X (Grade 3 & 4)^†^	(81)
Lima (2022)	51	X		X		(82)
Othman (2022)	88	X		X		(83)
Roth (2022)	121		X (Class I)		X (Grade ≥2)^†^	(31)
Schecter (2022)	34		X (Class ≥3)	X		(84)
Talwar (2022)	51	X		X		(85)

Unrestricted = “all comers” design; Restricted = defined by protocol inclusion criteria.

^†^ VHWG grade (original scale).

^‡^ VHWG grade (modified scale).

A subset of the identified publications (*n* = 12) involved *in vitro* studies, benchtop testing, cadaver studies, review articles, and *ex vivo* studies that were broadly categorized as “bench studies” as shown in Figure 2, and Table 1 (5–8, 22, 36–42). Several major themes were identified within these studies, namely a correlation between the mechanical strength of P4HB meshes and their remaining molecular weight during degradation studies, as well as the cellular response to P4HB mesh constructs and their degradation products. In an *in vitro* degradation test performed by Martin et al. (6), P4HB mesh exhibited a significant reduction in mechanical strength and molecular weight over time, which correlated with the integrity of the individual fibers comprising the mesh. They found that when changes to the structure of the mesh could first be appreciated visually through scanning electron microscopy, the mechanical strength and molecular weight of the P4HB mesh had already been reduced by approximately 90% (6). Martin et al., utilized this mechanical strength and molecular weight data to construct a standard curve that can be used to predict the ball burst strength of P4HB mesh when molecular weight is known. Care must be used, however, before applying such a correlation to different forms of the polymer, as the degree of orientation imparted during processing affects the susceptibility of P4HB to enzymatic degradation and surface erosion *in vivo*.

A number of bench studies also investigated the degradation products of P4HB mesh (i.e., 4HB monomer) using murine bone marrow-derived macrophages (36, 37, 42). These studies found that the degradation products are not cytotoxic, and in fact, may help promote bacterial clearance by macrophages through an upregulation of antimicrobial peptides (AMPs) (36, 37, 42). The degradation products have also been shown to influence the cellular expression of macrophage phenotypes of murine bone marrow-derived macrophages (37) and potentially provoke a unique polarization state in human peripheral blood-derived macrophages (41). Cellular response to P4HB meshes was favorable compared to polypropylene meshes, including higher collagen I : III ratio, as well as improved cellular attachment and proliferation (38, 40). Additionally, despite greater surface area relative to polypropylene mesh, P4HB meshes were not associated with greater bacterial adhesion or biofilm formation (39).

Fourteen publications identified by this scoping review involved preclinical studies in a variety of animal models and species, including pigs, rats, rabbits, sheep, mice, and vervets (Figure 2, and Table 1) (6, 23, 24, 26, 36, 37, 43–49, 89). Three of these studies (*n* = 3) contained both a benchtop component and a preclinical component (6, 36, 37). These studies are reported in both categories and included in Figure 2, and Table 1. Several outcomes were identified within these preclinical studies. First, P4HB meshes provided a durable and long-lasting hernia repair with mechanical strength of the mesh-repaired site equal to or greater than the native porcine abdominal wall in studies of 24-, 52- and 72-week duration (i.e., 6-, 12- and 18-month duration) (6, 43, 44). Second, P4HB meshes activated macrophages from a pro-inflammatory phenotype (M1) to a pro-remodeling phenotype (M2) earlier in the postoperative period than all of the other meshes evaluated (i.e., Bard™ Mesh, TIGR®, Bio-A®, and Strattice™ meshes). Finally, in numerous studies involving subcutaneous dorsal implantation of P4HB mesh with deliberate bacterial contamination, monofilament P4HB meshes exhibited significantly reduced bacterial colonization relative to other mesh types (36, 45, 89).

The majority of the identified publications (*n* = 50/79; 63%) involved clinical studies describing the use of P4HB mesh in plastic and reconstructive surgery (*n* = 10) (13–15, 18–21, 56, 61, 64) or general surgery applications (*n* = 40) (11, 12, 27–31, 50–55, 57–60, 62, 63, 65–85) as shown in Figure 2, and Table 1. In the area of plastic and reconstructive surgery, P4HB meshes have been utilized primarily in cosmetic breast applications (21) including mastopexy/reduction mammaplasty (18, 19), prevention of bulge at the donor site on the abdominal wall (14, 64), support of the lower pole (13, 20, 56), tissue expander-based reconstruction (15), and 3-D mesh breast reconstruction with autologous fat grafting (61).

P4HB meshes have been utilized extensively in general surgery for a wide variety of hernia repair and abdominal wall reconstruction applications (11, 12, 27–31, 50–55, 57–60, 62, 63, 65–85). A small number of studies (*n* = 2 in each group) evaluated P4HB mesh for incisional hernia prophylaxis (27, 54) and inguinal hernia repair (Table 1, and Table 2) (11, 28). Several studies (*n* = 5) also explored the use of P4HB mesh in hiatal/paraesophageal hernia repair (Table 1, and Table 2) (29, 57, 58, 79, 80). These studies reported favorable clinical outcomes, including low hernia recurrence rates (range: 0%–8%) and no surgical site infections (SSI), mesh-related complications, or reoperations with follow-up periods of at least 1 year.

Most of the general surgery studies identified by this literature review (*n* = 31/40; 78%) utilized P4HB mesh in the repair of ventral/incisional hernias, primarily at the midline (Table 1 and Table 3) (12, 30, 31, 50–53, 55, 59, 60, 62, 63, 65–78, 81–85). Primary fascial closure was planned in all cases, with definitive repair as the goal. In contaminated cases, bridging repair with P4HB was performed as an initial stage operation, with a future, planned definitive repair. Twenty-five (*n* = 25) studies utilized retrorectus/retromuscular, *n* = 11 underlay, *n* = 18 onlay, and *n* = 2 inlay mesh repair, with many studies incorporating a variety of surgical approaches and tissue planes. These studies documented a wide range of follow-up periods, including short-term (<2 years), intermediate (2–3 years), and long-term (>3 years) studies (Table 3), with P4HB meshes implanted in several different tissue planes, through a variety of surgical techniques. It should be noted that clinical outcomes were evaluated in patients along the entire continuum of Centers for Disease Control (CDC) wound classes (33) and Ventral Hernia Working Group (VHWG) grades (34, 35), ranging from clean cases at low risk of postoperative complications to contaminated/dirty cases at high risk of postoperative complications (Table 4). Systematic reviews (59), commentaries (86), and clinical studies of CDC Class I (clean) cases only (51, 65) comprised a small number of articles, with the majority of the identified clinical studies describing complex cases with elevated CDC wound class or comorbidities placing patients at high risk of postoperative complications (*n* = 13) (12, 30, 31, 50, 52, 55, 60, 62, 71, 75, 76, 83, 85) or “off-label” use of P4HB mesh to repair ventral/incisional hernias in potentially contaminated or contaminated fields (*n* = 15) (11, 53, 63, 66–70, 72–74, 77, 78, 81, 82, 84). After a thorough evaluation of the clinical studies identified by this scoping review, several major themes emerged, namely: (1) P4HB mesh provides long-term strength at the repair site, leading to acceptable rates of recurrence as compared to higher-risk cohorts and those repaired with non-synthetic biomaterials (30, 31, 66, 70, 75, 76); (2) P4HB mesh performs favorably in contaminated settings where permanent synthetic mesh use may be higher risk or contraindicated, resulting in low incidence of surgical site infection (SSI) (70, 76, 90); and (3) P4HB mesh represents an alternative for ventral/incisional hernia repair relative to biologic meshes and when permanent synthetic mesh complications are taken into consideration (30, 69).

P4HB mesh provides a long-term resorption profile with an initial mechanical strength similar to permanent synthetic mesh (6). However, once the P4HB polymer has been fully resorbed, the mesh contributes negligible strength to the overall repair, allowing for the potential for hernia recurrence. The current scoping review documented comparable recurrence rates for P4HB mesh at 3 and 5 years postimplantation [6.6%–17.9% (66, 70, 75, 76) and 12.9%–22.0% (30, 31), respectively] as permanent synthetic mesh at 5 years postimplantation (12.7%) (91) in study populations involving a variety CDC wound classes and VHWG grades.

The clinical literature also revealed that P4HB mesh performs favorably in the setting of contamination, evidenced by low rates of SSI at 3 years postimplantation. Similar rates were reported in studies involving all CDC wound classes and VHWG grades (i.e., 5%–7%) (70, 90). These outcomes are supported by the bench and preclinical studies in which P4HB mesh degradation products were shown to promote bacterial resistance and tissue remodeling through upregulation of AMPs and influence over macrophage phenotype (36, 37). In CDC class I (clean) wounds, P4HB was associated with 9.3% SSI, a rate higher than seen in mesh based cohorts (76). However, permanent synthetic meshes have been associated with higher rates of SSI in studies involving CDC wound classes II–IV [e.g., 7%–19% SSI at 30 days postimplantation in studies involving CDC wound classes II–III (92) and 14% SSI at a median follow-up of 24 months in studies involving CDC wound classes II–IV (93)].

Studies demonstrated a cost savings associated with P4HB mesh relative to biological tissue-based materials such as porcine dermal matrix (Strattice™, Allergen Aesthetics, Madison, NJ) (30, 69). In a study by Buell et al*.*, P4HB mesh exhibited improved clinical outcomes including hernia recurrence and was associated with a cost savings of $10,595 compared to porcine dermal matrix (Strattice™, *p* = 0.005) (30). In a cost-effectiveness model, Charleux-Muller et al., showed that P4HB mesh repairs are associated with fewer serious complications (21% vs. 33%) and are less costly than biological tissue-derived materials (cost savings of $42,883) in VHWG Grade 3 hernia repairs (69).

## 4. Discussion

Poly-4-hydroxybutyrate (P4HB) mesh has been studied for more than a decade, including a broad collection of *in vitro*, preclinical, and clinical studies. To date, this biomaterial has been utilized in patients along the entire spectrum of CDC wound classes and VHWG grades, ranging from patients with clean wounds at low risk of postoperative complications to patients with contaminated/dirty wounds at high risk of postoperative complications. Short-term, intermediate, and long-term clinical studies have been performed, including implantation in many different tissue planes, using a wide variety of surgical techniques. This comprehensive literature review yielded 79 peer-reviewed, published studies evaluating P4HB mesh in a wide variety of bench, preclinical, and clinical study designs. Importantly, 40 clinical studies provide a current understanding of the performance of P4HB mesh in hernia repair and abdominal wall reconstruction. In the scope of hernia repair, P4HB remains a novel material. These early studies suggest that P4HB mesh provides strength at the repair site beyond its resorption profile, leading to reasonable rates of recurrence especially in settings where use of permanent synthetic mesh is avoided or contraindicated. P4HB performed favorably in the setting of contamination, resulting in low incidence of surgical site infection (SSI). Recent long-term studies, including Roth (31) and Talwar (85), also demonstrated improvement in long-term quality of life metrics.

Preclinical studies demonstrate long-term resorption of P4HB over a period of approximately 12–18 months, during which the mesh loses all of its mechanical strength (6, 43, 44, 47). The clinical studies identified in this literature review provided insight into the performance of P4HB in a variety of patient populations and clinical scenarios. Once the P4HB polymer has been fully resorbed, the mesh no longer contributes to the mechanical strength of the repair. This property has important implications in hernia repair, stressing the importance of operative technique and primary fascial closure to achieve durable long-term results. Two studies utilized P4HB mesh in the inlay position, serving primarily as tissue coverage and to prevent fascial retraction as a salvage or staged operation for future definitive repair. In all clinical studies where definitive hernia repair was desired, the defect was closed with mesh either in the underlay, retrorectus/retromuscular plane, or onlay position. These findings compliment the premise that the long-term resorbable matrix confers mechanical strength during the vulnerable phases of wound healing, shifting the curve to favorable mature scar formation with the potential for a durable repair beyond the resorption profile of the P4HB.

Several clinical studies warrant in-depth discussion. First, in a study restricted to patients with CDC class I (clean) wounds at high risk of developing postoperative complications (i.e., VHWG grade ≥2) Roth et al. evaluated clinical outcomes including SSI, hernia recurrence, and quality of life over a period of 5 years (31, 52, 76). Incidence of SSI remained consistent over time with 9.0%, 9.3%, and 10.1% SSI reported at 18, 36, and 60 months, respectively (31, 52, 76). Throughout the follow-up period, recurrence rates rose from 9% at 18 months to 17.9% at 36 months, and ultimately, 22% at 60 months postimplantation (31, 52, 76). However, these reported recurrence rates are interesting given that at this long-term time point, it is expected that there is no residual material and can be viewed as acceptable given a patient population at high risk of postoperative complications such as recurrence (i.e., VHWG grade ≥2). In this context, P4HB is a viable option, especially where avoidance of a permanent synthetic is desired based upon patient factors, operative conditions, and both patient and surgeon preference. Kanters et al. have demonstrated that the risk of hernia recurrence increases significantly in patient populations with elevated VHWG grade (35). In this context, long-term recurrence rates for P4HB mesh are comparable to those reported for permanent synthetic meshes at 5 and 10 years [12.7% (91) and 32% (94), respectively].

In a study involving patients across all CDC wound classes and all VHWG grades, Buell et al. evaluated long-term clinical outcomes associated with P4HB mesh compared to a porcine dermal matrix (Strattice™) (30). At 60-months postimplantation, Strattice™ was associated with approximately 3× greater incidence of recurrence (38.1%) and SSI (31.0%) compared to P4HB (12.9% recurrence, *p* = 0.017; 12.9% SSI, *p* = 0.071) (30). Hernia recurrence and SSI rates associated with P4HB were comparable to the published literature.

In another study, P4HB mesh was utilized “off-label” in contaminated hernia repairs, Christopher et al*.* reported hernia recurrence of 8.3% and SSI of 16.7% at 24 months postimplantation in a patient population with CDC wound class ≥2 and VHWG grade ≥3 hernia repairs (72). These represent favorable results compared to similar studies involving other types of hernia repair materials. In the COBRA trial, Rosen et al. reported comparable SSI (18%) and 2× higher recurrence rates (17%) for another fully resorbable polymer mesh material (Bio-A®, W.L. Gore & Associates, Inc., Flagstaff, AZ) implanted in clean-contaminated and contaminated wounds in a population with multiple comorbidities predictive of postoperative complications (95). In the RICH trial, Itani et al*.* reported rates of recurrence of 28% and SSI of 35% for a biological tissue-derived material (Strattice™) in patients with CDC class II, III, or IV wounds (96).

Finally, when P4HB mesh was utilized to replace infected mesh in a single-stage approach, Bueno-Lledo et al., reported a recurrence rate of 6.6% and SSI of 3.3% (mean follow-up of 36.5 months) (66). They compared these results to a second cohort in which permanent synthetic mesh was used to replace infected mesh in a two-stage approach. The permanent synthetic mesh cohort exhibited a recurrence rate comparable to P4HB mesh (7.2%; *p* = 0.101), but 3× greater incidence of SSI (9.8%, *p* = 0.002) (66).

Previous studies have shown that the risk of surgical site occurrences (SSOs) such as SSI increases with increasing CDC wound class (35). However, the current literature review showed that P4HB mesh is associated with relatively low rates of SSI compared to other resorbable materials or biological tissue-derived matrices, including in patient populations with elevated CDC wound class. These outcomes are supported by bench and preclinical studies in which P4HB mesh degradation products were shown to promote bacterial resistance and tissue remodeling through upregulation of AMPs and influence over macrophage phenotype (36, 37). Based on the current literature, P4HB provides a viable option for repair in this challenging patient population at higher risk for infection and recurrence.

As a scoping review, the current study is not without limitations. First, the quality of the studies included in this review was not formally assessed as would be completed in a systematic review or meta-analysis. Nor, were the potential biases of any of the included studies discussed. The majority of the studies in this review are observational with the corresponding limitations. Roth et al. provides the longest-term follow-up to 60 months, but is a single-arm, observational trial with follow-up capturing only 44.6% of the cohort (31). Due to the limited follow-up, the actual recurrence and SSI rates would be expected to be higher, and the risk of bias is greater than data derived from a randomized controlled trial. This is an inherent limitation and challenge of large-scale clinical research and provides insight to guide future research.

Several knowledge gaps in the existing literature were identified by the current scoping review, generating a number of research questions to be addressed. Future studies should continue to evaluate P4HB mesh for a variety of clinical applications in the field of hernia repair. Inguinal hernia repair and incisional hernia prophylaxis are particularly promising areas of research that warrant additional studies with long-term follow-up and comprehensive evaluation of clinical outcomes. Future studies are also needed to compare outcomes associated with P4HB mesh to those of permanent synthetic meshes, particularly in non-complicated cases (i.e., CDC class I, VHWG grade 1). Permanent synthetic meshes have traditionally been utilized in these cases, and data is currently lacking for P4HB mesh in this patient population. The operative technique and mesh position are key to obtaining a durable repair with a completely resorbable mesh. Dedicated studies and a systematic review are needed to clearly define the optimal surgery, tissue plane, and the significance of fascial closure. One of the advantages of a fully resorbable material such as P4HB is to address increasing public sentiment and medicolegal implications regarding the use of permanent prosthetics in surgery and provide durable alternatives. The ongoing desire to identify the optimal prosthetic in hernia repair remains one of the central research themes in the field of hernia and abdominal wall reconstruction. Finally, randomized controlled trials (RCT) evaluating P4HB mesh against other biomaterials are absent in the scientific literature and are needed to properly delineate the appropriate and ideal role of P4HB in hernia repair.

## 5. Conclusions

The use of P4HB is safe and effective in hernia repair, but more research is needed. Repair with P4HB provides strength beyond the scope of its resorption profile with promising results in the context of contamination and demonstrates a role where the avoidance of a permanent mesh prosthesis is desired. Future studies are warranted to evaluate the appropriate and optimal role for P4HB in hernia and abdominal wall surgery, including hernia prophylaxis, inguinal hernia, and as an alternative to permanent synthetic mesh in routine hernia repair.

## Data Availability

The original contributions presented in the study are included in the article/Supplementary Material, further inquiries can be directed to the corresponding author.
